# A multiband SSr diode RF rectifier with an improved frequency ratio for biomedical wireless applications

**DOI:** 10.1038/s41598-023-40486-x

**Published:** 2023-08-15

**Authors:** Surajo Muhammad, Mohamed Ibrahim Waly, Nasser Ali AlJarallah, Ridha Ghayoula, Ahmed S. Negm, Amor Smida, Amjad Iqbal, Jun Jiat Tiang, Mardeni Roslee

**Affiliations:** 1 Faculty of Engineering, Centre For Wireless Technology (CWT), Multimedia University, Cyberjaya, 63100 Malaysia; 2 Department of Medical Equipment Technology, College of Applied Medical Science, Majmaah University, Al Majmaah, 11952 Saudi Arabia; 3 Department of Biomedical Engineering and System (formerly) Higher Institute of Engineering-El Shorouk Academy, Cairo Governorate, Egypt; 4 Department of Business Administration, Majmaah University, 11952 Majmaah, Saudi Arabia; 5 AlMaarefa University, Riyadh, Saudi Arabia; 6 Department of Electrical and Computer Engineering, Laval University, Quebec City, QC G1V0A6 Canada; 7 Microwave Electronics Research Laboratory, Faculty of Mathematical, Physical and Natural Sciences of Tunis, Tunis El Manar University, Tunis, 2092 Tunisia; 8 Consultant, Saudi Consolidated Engineering Company Healthcare Technology Management Administration, King Fahad Medical City, Riyadh, Saudi Arabia; 9 Institut National de la Recherche Scientifique (INRS), Montréal, QC H5A1K6 Canada; 10 Department of Network and Communications Engineering, Al Ain University, 64141, Al Ain, UAE; 11 Department of Electronics and Telecommunication Engineering, Ahmadu Bello University, Zaria, 810211 Nigeria

**Keywords:** Biomedical engineering, Electrical and electronic engineering

## Abstract

This paper described a four-band implantable RF rectifier with simplified circuit complexity. Each RF-rectifier cell is sequentially matched to the four operational frequencies to accomplish the proposed design. The proposed RF rectifier can harvest RF signals at 1.830, 2.100, and white space Wi-Fi bands between 2.38 to 2.68 GHz, respectively. At 2.100 GHz, the proposed RF harvester achieved a maximum (radio frequency direct current) RF-to-DC power conversion efficiency (PCE) of 73.00% and an output DC voltage $V_{DC}$ of 1.61 V for an RF power of 2 dBm. The outdoor performance of the rectenna shows a $V_{DC}$ of 0.440 V and drives a low-power bq25504-674 evaluation module (EVM) at 1.362 V. The dimension of the RF-rectifier on the FR-4 PCB board is 0.27$\lambda _{g}$ $\times$ 0.29$\lambda _{g}$. The RF-rectifier demonstrates the capacity to effectively utilize the frequency domain by employing multi-band operation and exhibiting a good impedance bandwidth through a sequential matching technique. Thus, by effectively controlling the rectenna’s ambient performance, the proposed design holds the potential for powering a range of low-power biomedical implantable devices. (BIDs).

## Introduction

**Figure 1:**
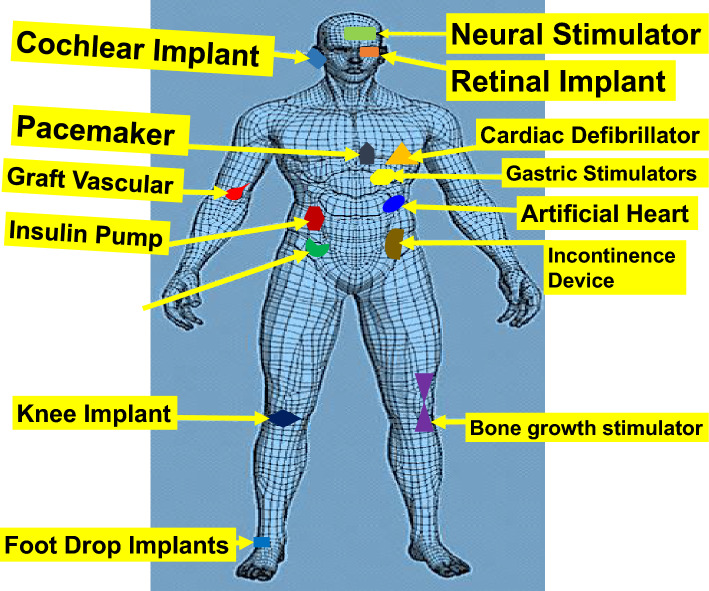
Various implantable medical devices and their physical location inside human body.

Low-power embedded devices are becoming increasingly popular in a variety of consumer and industrial applications^1,2^. Biotelemetry, drug proportion, and allocation are some of the impacts BIDs technology made in the healthcare profession^3,4^. Recent advancements in materials and manufacturing have produced a novel, softer, adaptable devices with lower-impedance electrodes^5–7^. Since the technology’s inception, traditional batteries and physical interconnecting cables have been employed in these implants^4,8^. Because of their short lifespan, the batteries must be revised after a single installation. However, a patient must undergo an uncomfortable, expensive, and unpleasant procedure to change these batteries^6,7,9^. Additionally, the interconnecting wires are unsafe and can lead to other infectious diseases^10^. Several techniques have been established to extract energy from various sources, including vibration, acoustic, sound, light, pressure, and heat, to address these issues and constraints^11–15^. The radio frequency (RF) waves can be harnessed to operate electrical components and replenish the implant’s batteries^8^. Thus, ambient electromagnetic (EM) sources are becoming more popular for powering biomedical implants^16^. It is crucial to harvest these sources because of the relatively low signal amplitude and the significant power needed to drive biomedical implants^17^. A configurable wireless power transmitter (WPT) can simply be employed as a signal source in implants when potential operating power is desirable^18^. The WPT can extend the battery’s lifetime of the implantable medical devices (IMDs) and ease patients’ pain during surgery^8,19^. Several studies on the WPT’s, such as microwave radiation and near field coupling^16,17,19^, have been introduced based on the various application condition. The near field coupling mechanism often outperforms the microwave radiation in terms of the transmission range, but is larger in size^12,18^. Thus, a potential mechanism for the miniaturized IMDs is WPT-based microwave radiation^16,17^. Figure 1, demonstrate how a WPT depends on the coupling between the transmitter and receiver. The WPT microwave receiving segment requires an implanted antenna and RF rectifier to receive and transform the radiated RF signal into a DC source^7^. The regulated characteristics of the transmitter have made the WPT evolve as a potential solution to the traditional ambient sources for charging and powering the implants^14,16^. A power source and a rectenna make the WPT system. Thus, biomedical implants stand to benefit significantly from RF energy harvesting due to its various advantages: wireless power transmission, prolonged battery lifespan, miniaturization capabilities, improved dependability, scalability, and environmental sustainability^2,18^. These benefits lay the groundwork for enhanced and patient-centric implantable medical devices, propelling healthcare advancements and elevating patients’ quality of life.

Most WPT commercial transmitters have preset signal intensities; the rectenna side need to be designed to capture the least amount of available signal efficiently. Thus, to efficiently harvest a range of accessible sources, an efficient rectifier is required. The authors in^20^ demonstrate a 0.915 GHz mm-sized rectenna for a deep-brain stimulation mechanism. The radiated energy from the source is received using interconnected rectenna elements. At an input power of 30 dBm, the implant harvester reported a highest efficiency is 59.70%, at the cost of a limited bandwidth (BW). The authors in^21^ and^22^ describe a relatively compact planar inverted-F (PIFA)-based implantable rectenna. The radiating element is achieved through capacitive loading by introducing a short-pin in^22^ and spiral patches in^21^. A wireless power link comprising an implantable rectifier is investigated in^22^. The findings demonstrate that the approach achieved higher DC power at the expense of a narrow-BW and high $P_{in}$. The authors in^23^ uses an external WPT to deliver power to an implantable battery free cardiac pacing. The design realizes an efficiency at a significant power of 12 dBm. A dual-band (2.45 and 5.8 GHz) RF rectifier is deployed for WPT energy harvesting (EH) in^24^. The design utilizes a HSMS2860 diode for a high input power source. At an input power of 12.0 dBm, the highest efficiency of 63.0% and 54.80% is realized. A 2.32 and 3.48 GHz Class-F mid-field system comprising a single-shunt diode is demonstrated by the authors in^25^. An implantable triple-band stacked single-shunt rectifier with a radial construction is introduced by the authors in^26^. Only one specified frequencies can be deployed for WPT’s, wake-up and sleep controllers, and data telemetry. The authors in^27^ present a triple band rectifier for biotelemetry applications at white space Wi-Fi band. The designed is applied to realized a 390 mV wirelessly and achieved 48.00%, 52.00%, 45.00% RF-to-DC PCE at -5 dBm. The authors in^28^ presents a triple band (1.95, 2.70, and 5.80 GHz) rectifier through multi-stepped transmission lines for WPT and RF energy harvesting (RFEH). The large electrical size described in the design increases the overall size of the implants. The studies showed that the technique can deliver peak efficiency of 62.2%, 59.40%, and 48.9% at an input power of 0 dBm. The researchers in^29^ described their work concerning a two-port, five-band RF rectifier. The physical dimensions of the rectifier were measured to be 75 mm $\times$ 75 mm, and the experimental results indicated that it attained an average power conversion efficiency of 23.2% when subjected to an input power of -20 dBm. The authors in^30^ and^31^ introduced a quad-band and six-band RF harvester. This system demonstrated a PCE of 15% and 67% when subjected to an input power of -20 and -5 dBm, respectively. However, the reported -10 dB operational bandwidth was relatively narrow across all six and fourth operating frequencies. The use of a single dual-diode was found to increase the rectifier parasitic capacitance at the diode junction. At the same time, the presence of sixth-order lumped elements MN within the four and three cell branches in^30^ and^31^ contributed to a reduction in the overall PCE due to parasitic effects. The researchers in^32^ presented a seven-band rectifier design operating at (1.80, 2.10, 2.40, 2.60, 3.50, 4.90, and 5.80 GHz). The rectifier was implemented using an SMS7630 diode. This work achieved a peak PCE of 64% when exposed to 4 dBm input power. The rectifier design achieved peak PCE at high power and a large dimension. However, there is limited harnessing of available RF power at frequencies of 3.50, 4.90, and 5.80 GHz. Also, most of the rectifiers that have been studied in the literature between^20^ and^25^ use either a single or dual operating frequency ($f_{o}$) with a narrow band. Besides the intricate circuitry and significant power is expected for the circuit to function in the implants efficiently. The large electrical size lowers the overall efficiency of the designs shown by the authors in^26–31^, and^32^ for low power application.

This paper proposes an implantable multiband RF rectifier for microwave WPT operating at 1.830 and 2.100 GHz and a broad white space ISM band (2.380 - 2.80 GHz). The 2.45 GHz ISM band was chosen because it is among the license-free bands. The proposed design is established around an L-shunt $\lambda _{g}$/8 MN via an impedance transformer (ITx) between the four-unit cells. The rectifier’s operating frequencies are systematically matched to the four cells, accordingly. The proposed design improves the operational BW and size by positioning the L-shunt between a series inductor and a radial shunt stub. This keeps the capacitance at the branch of the rectifier to a minimum, which improves the overall PCE of the RF rectifier. Hence, the proposed design demonstrates a unique sequential matching technique that simplifies the circuit complexity over a wide range of harvested frequencies, high PCE, good $V_{DC}$ capability, compact size, and applicability to power low-power biomedical implantable devices and is a promising option for wireless power transfer (WPT) than single-band WPT systems.

## Rectifier design

**Figure 2:**
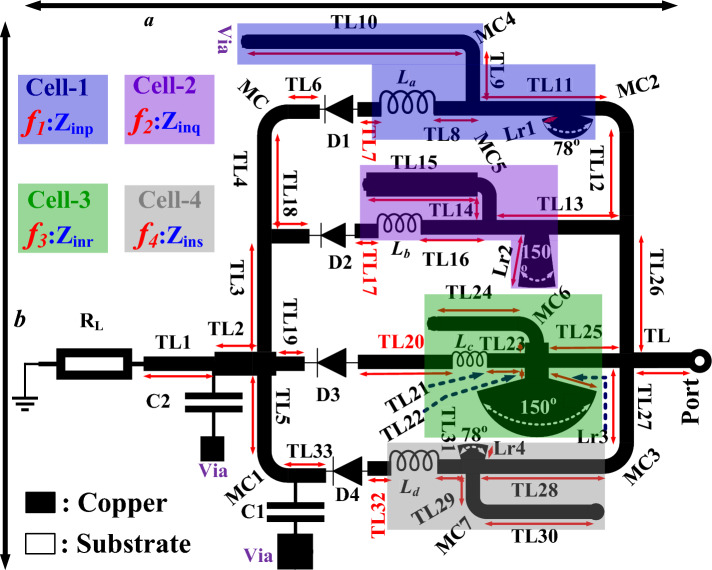
EM model layout of the proposed wideband RF-rectifier.

The proposed technique is implemented across the four operating frequencies using a unique architecture shown in Fig. 2. Cell-1, Cell-2, Cell-3, and Cell-4 are the four parts of the design, each configured with an L-shunt MN, a radial stub, and a series inductor. The proposed MN is employed to convert the rectifying unit’s (RU) complex impedance into the source impedance by ITx matching TL7(TL8), TL17(TL16), TL20(TL21), and TL32(TL31). Three sets of line connections (TL9-TL10), TL11, and a radial stub of length Lr1 across D1 are used to realize the MN in Cell-1. To match D2, an L-section (TL14-TL15) MN was introduced in Cell-2 with the help of TL13 and the Lr2 stub. Cell-3 comprises an L-section (TL25-TL24), TL25, and an Lr3 stub for matching D3. Whereas D4 matching is achieved using L-sections TL29-TL30, TL28, and the Lr4 stub. Both sections are implemented around a single series (SSr) diode configuration. A suitable rectifying diode evaluation for transformation is one of the most crucial parts of establishing the RF-rectifier for extracting the RF signal. This design is based on the HSMS2850 Schottky diodes D1, D2, D3, and D4. It was made using the small-outline transistor (SOT)-323 circuit layout method. The SOT-323 approach of circuit architecture was used in its manufacturing process. Because of its low junction capacitance of 0.18 pF and low sensitivity of 150 mV, the diode is a potential device for low-power applications. The SSr diodes are coupled to an inductor and a radial stub in the proposed design, allowing for low-power operation over a broad frequency range (Table 1). The use of the short circuited-stubs allows for a more smooth flow of DC into the circuit.

**Table 1:** Specifications and attiributes of the proposed rectifier.

Parameters	Width/Length (mm)	Parameters	Width/Length (mm)	Parameters	Width/Length (mm)
TL	0.6/1.5	TL12	0.6/3.5	TL24	0.6/3.4
TL1	0.6/3.0	TL13	0.6/5.0	TL25	0.6/3.75
TL2	1.0/1.3	TL14	0.6/1.0	TL26	0.6/5.5
TL3	0.6/5.8	TL15	1.0/4.7	TL27	0.6/3.85
TL4	0.6/3.5	TL16	0.6/3.4	TL28	0.6/5.5
TL5	0.6/3.65	TL17	0.6/1.0	TL29	0.6/1.5
TL6	0.6/1.6	TL18	0.6/1.6	TL30	0.6/4.6
TL7	0.6/1.0	TL19	0.6/1.3	TL31	0.6/1.5
TL8	0.6/1.5	TL20	0.6/4.0	TL32	0.6/1.0
TL9	0.6/2.0	TL21	0.6/2.0	TL33	0.6/1.6
TL10	0.6/9.0	TL22	1.0/0.8	*b/a*	20.7/19.3
TL11	0.6/5.5	TL23	1.0/0.7	Lr1/Lr2	0.6/2.2

Parameters	$W_c$/R (mm) ($\theta$ = 90$^o$)	Parameters	Values/Values (nH)	Parameters	Values/Unit
MC	0.6/1.0	$L_{a}$/$L_{b}$	1.0/1.0	Lr3/Lr4	2.1/0.6
MC4	0.6/0.5	$L_{c}$/$L_{d}$	5.1/5.1	C1 *(pF)*/$R_{L}$(k$\Omega$)	330/5

[Microstrip curve bend (MC) = W$_c$, R, $\theta$], [Transmission line (TL) = W, L], [Radial stub length = Lr].

MC = MC1 = MC2 = MC3 = MC6, MC4 = MC5 = MC7, C1 = C2

Impedance Transformer (IT$_x$) MN: TL7, TL17, TL20, and TL32.

(All units are in mm, Diagram not up to scale).

An analytical study was conducted on the matching performance to ensure the proposed model functions optimally. The optimal performance of the pre-design RF-rectifier is then estimated using a source pull simulation in an advance design system (ADS). With the pre-design analysis’s help, the load’s ($R_{L}$) the capacitor’s (C) appropriate size and can be inspected. The proposed topology performs better when the power level across each cell is between -5 and 2 dBm. At a 0 dBm input power level, the equivalent impedances for Cell-1, Cell-2, Cell-3, and Cell-4 are presented in Table 2. The preliminary design demonstrates an improvement at 330 pF and 5 k$\Omega$. Each cell is designed for a particular frequency range: Cell-1 ($f_{1}$ = 1.83 GHz), Cell-2 ($f_{2}$ = 2.1 GHz), Cell-3 ($f_{3}$ = 2.45 GHz), Cell-4 ($f_{4}$ = 2.66 GHz). It is important for the MN to keep the imaginary side of each diode stable across the frequencies $f_{1}$, $f_{2}$, $f_{3}$, and $f_{4}$. This keeps the impedance mismatch of $Z_{inp}$, $Z_{inq}$, $Z_{inr}$, and $Z_{ins}$ in the RU to about 50 $\Omega$.

First, an L-shunt MN connected to TL7 is modeled in the architecture of Cell-1’s impedance matching network (IMN) at $f_{1}$. The Upper ($f_{1u}$) and lower ($f_{1l}$) frequencies are related in a complex conjugate form by the TLs ((TL9-TL10), TL11), to match the admittances at the common node. Hence, $Z_{9}$ $|$ $f_{1l}$ = [$Z_{9}$ $^{*}$ $|$ $f_{1u}$], $Z_{11}$ $|$ $f_{1l}$ = [$Z_{11}$ $^{*}$ $|$ $f_{1u}$], $Z_{8}$ $|$ $f_{1l}$ = [$Z_{8}$ $^{*}$ $|$ $f_{1u}$]. At a particular operating frequency, in the direction of the load where the RF signal is being cut off by the DC pass filter (DPF), the impedance should be infinite. Therefore, $Z_{9}$ $|$ $f_{1l}$ = [$Z_{9}$ $^{*}$ $|$ $f_{1u}$] = 0 The TL7 parameters ($\theta _{7}$ and $Z_{7}$) change the odd imaginary part of $Z_{7}$ into a symmetrical form and keep the real part in balance.

**Table 2:** Corresponding input impedance at 0 dBm input power for the diodes D1, D2, D3, and D4 without the MN.

$Z_{inp}$ @ $f_{1}$	$Z_{inq}$ @ $f_{2}$
79.25 - *j*249.50	58.47 - *j*246.50
$Z_{inr}$ @ $f_{3}$	$Z_{ins}$ @ $f_{4}$
36.00 - *j*194.63	33.01 - *j*172.24

Assume that the values $Z_{inp}$ at $f_{1l}$ and $f_{1u}$ are governed by: $Z_{p1}$ $|$ $f_{1l}$ = $R_{Lp1}$ $+$ $X_{Lp1}$ and $Z_{p1}$ $|$ $f_{1u}$ = $R_{Lp2}$ $+$ $X_{Lp2}$. Then:

1 $$\begin{aligned} Z_{inp}(f_{1l})&= Z_{p1}\Big [{\frac{(R_{Lp1}+jX_{Lp1})+jZ_{p1}\tan \theta _{p1}}{Z_{p1}+j(R_{Lp1}+jX_{Lp1})\tan \theta _{p1}}}\Big ] \end{aligned}$$

2 $$\begin{aligned} Z_{inp}(f_{1u})&= Z_{p1}\Big [{\frac{(R_{Lp2}+jX_{Lp2})+jZ_{p1}\tan \theta _{p1}}{Z_{p1}+j(R_{Lp2}+jX_{Lp2})\tan \theta _{p1}}}\Big ] \end{aligned}$$

where $\theta _{p1}$ denotes for the electrical length of the line at $f_{1l}$ and $f_{1u}$. These frequencies are controlled by a frequency ratio (*e*), such that $f_{1l}$ $f_{1u}$. Therefore, $f_{1u}$ = *e* $\times$ $f_{1l}$. Thus, $\theta _{p1}$ $(f_{1u})$ = *e* $\times$ $\theta _{p1}$ $(f_{1l})$ = $e\theta _{p1}$. The complex conjugate of $Z_{inp}$ becomes $Z_{inp}$ $|$ $f_{1l}$ = {$Z_{inp}$ $^{*}$ $|$ $f_{1u}$} through the TL7 transformation. The admittance (Y$_{p1}$) is then conjugated using the derived parameters $Z_{p1}$ and $\theta _{p1}$.

3 $$\begin{aligned}&Z_{7} = \big \{{(R_{Lp1}R_{Lp2}+X_{Lp1}X_{Lp2})+(X_{Lp1}+X_{Lp2}) \big (\frac{R_{Lp1}X_{Lp2}-R_{Lp2}X_{Lp1}}{R_{Lp2}-R_{Lp1}}}\big )\big \}^{\frac{1}{2}} \end{aligned}$$

4 $$\begin{aligned}{}&\begin{aligned} \theta _{7}=&\frac{1}{(1+e)}\Big [\arctan \Big (\frac{Z_{p1}(R_{Lp1}-R_{Lp2})}{(R_{Lp1}X_{Lp2}-X_{Lp1}R_{Lp2})}\Big )+d\pi \Big ],\quad \\&\qquad \qquad \qquad \qquad \qquad \text {for } d = 0, 1, 3, ... \end{aligned} \end{aligned}$$

*d* is an integer chosen to improve the design process. Both $Z_{7}$ and $\theta _{7}$ are calculated to have impedances of 56.86 $\Omega$ and 61.85$^o$, using Eqs. (3 and 4), respectively, when d = 3. At first, $Z_{inp}$, $Z_{inq}$, $Z_{inr}$, and $Z_{ins}$ were used in calculating the pre-design model in ADS, to determine the values of the ITx (TL7, TL17, TL20, and TL32), as shown in Table 3. TL7 is further segmented into the TL8 with the addition of an inductor ($L_{q}$) coupled to the ITx. The concept is applied between (TL17 and TL16), (TL20 and TL21), and (TL32 nad TL31). Inductive lumped components have been added to the two parts to boost the RF signal received by the RU with minimal TL length and loss. On the other hand, the TL11 at $f_{1l}$ and $f_{1u}$ exhibits the conjugated connection found in TL7. This is attained if TL7’s ($\theta _{11}$) at $f_{1l}$ satisfies the condition in Eq. (5).

5 $$\begin{aligned} \theta _{11} = \pi \times \frac{1}{[1+d]} \end{aligned}$$

The conjugate matching between the two frequencies generates TLIN admittances. Hence, at the source terminal, the impedance $Z_{s}$ requires that the real element of Y$_{11}$ be equal to that of Y$_{7}$ via TL7; consequently:

6 $$\begin{aligned} Z_{11} = \sqrt{\Big [{\frac{Z_{s}(1+G_{7}Z_{s}+\tan ^{2}\theta _{11})}{G_{7}\tan ^{2}\theta _{11}}}\Big ]} \end{aligned}$$

where $G_{7}$ stands for $Y_{7}$’s real element. Eqs. (5 and 6) show that, for frequencies $f_{1l}$ and $f_{1u}$, $Z_{11}$ and $\theta _{11}$ are 85.25 $\Omega$ and 20.62$^o$, respectively.

**Table 3:** Analytical ITx values between Cell-1 to Cell-4.

TL7 : {$Z_{7}$, $\theta _{7}$}	TL17 : {$Z_{17}$, $\theta _{17}$}
{91.86 $\Omega$, 61.85$^o$}	{92.1, 80.43}
TL20 : {$Z_{20}$, $\theta _{20}$}	TL32 : {$Z_{32}$, $\theta _{32}$}
{96.10, 116.00}	{95.12, 111.32}

The radial stub Lr1 and TL9 make up for $Y_{inp}$’s imaginary impedance, keeping the real impedance at $Y_{in7}$ = $1/Z_{in7}$. Therefore, the TL9 is represented by A($Z_{9}$, $\theta _{9}$ at $f_{1l}$) and $e \theta _{9}(f_{1l})$. In addition, the entire short-circuited L-shunt stub is guided by $Y_{in9}(f_{1l})$ = $1/jZ_{9} \tan e\theta _{9}(f_{1l})$. It is desirable for the admittance of TL9 to be odd-symmetric at $f_{1l}$ and vary inversely with the imaginary part of $Y_{in7}$.This effectively reduces the imaginary part to nearly zero. Loading TL7 into *G* + *jB* and *G* - *jB* conjugates at $f_{1l}$ and $f_{1u}$ transforms these admittances.

The short-circuited L-shunt stub’s admittance may then be enhanced with the following:

7 $$\begin{aligned} Y_{in9} = {\left\{ \begin{array}{ll} \frac{1}{(jZ_{9}\tan \theta _{9})} = jB_{9}, &{} \text {at}\ f_{1l},\\ \frac{1}{(jZ_{9}\tan (t\theta _{9}))}=-tB_{9}, &{} \text {at}\ f_{1u}. \end{array}\right. } \end{aligned}$$

From Equation 7:

8 $$\begin{aligned} Z_{9} = \frac{1}{(B_{9}\times \tan (e\theta _{9}))} \end{aligned}$$

where:

9 $$\begin{aligned} \theta _{9} = \frac{\pi }{(1+e)} \end{aligned}$$

Equations (8 and 9) calculate $Z_{9}$ and $\theta _{9}$ to be 95.12 $\Omega$ and 67.40$^o$, respectively. Selecting the appropriate $Z_{i}$ and $\theta _{i}$ throughout the design process helps prevent uncontrolled and pointless characteristic impedance.

Several stubs comprising linked microstrip curve bend (MCURVE), also known as MC, radial stubs, and stepped-impedance lines, are used in ADS to reduce manufacturing limitations and enhance design performance. The TL9 is then partitioned into the TL10 through MC4. Also, a radial stub Lr is integrated into the susceptance block TL9 to enhance the design’s operational BW. The stub, whose Lr1 is set at 0.6 mm and optimized with a 78$^o$ bend angle, gives the design an extra degree of freedom.

Similar to how Cell-1’s parameters were modeled, Cell-2 (T17-TL16, TL13, TL8, TL15-TL14, and MC5), Cell-3 (TL20-TL21, TL25, TL24-25, MC6), and Cell-4 (TL31-TL232, TL28, TL29-30, MC7) parameters were also established by using Eqs. (1 to 9). A series of inductive matchings ($L_{a}$, $L_{b}$, $L_{c}$, and $L_{d}$) are deployed towards the diode’s anode and between the ITx, across the four cell blocks. The first designs of $L_{a}$, $L_{b}$, $L_{c}$, and $L_{d}$ were made using the ideal palette in ADS at 1.5, 1.7, 4.5, and 6 nH, respectively. The inductors were adjusted between 0.5 and 10 nH to make up for the effect of the transmission line on the design. More optimization was then applied to the proposed circuits. A relevant *muRata* components from the ADS library was added to replace the elements of the ideal inductor. The low-power LQP03TG1N0B02 and LQP03TG5N1H02 series model with 0603 layouts is used for optimal inductor performance in the design, as depicted in Fig. 1. In order to reduce the amount of interference in the circuit, the capacitor C is used to “shunt” the diode D4. The two capacitor filters from *muRata* have the model number GCH1555C1H331JE01 from the 0402 families.

A 1.60 mm thick FR-4 substrate (tangent loss = 0.02 and dielectric constant = 5.6) was used as the basis for the design. The proposed implantable RF rectifier has integrated TL3, TL4, TL5, TL12, TL26, and TL28 to improve the design architecture’s functionality. A 50 $\Omega$ TL terminates the four cell block configurations. This is followed by optimization and fine-tuning of the complete RF-rectifier unit, as presented in Fig. 2. The specifications and attributes of the proposed RF rectifier is reported in Table 1.

**Figure 3:**
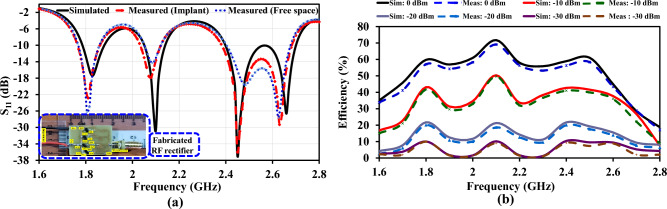
The proposed design’s simulated and measured: (**a**) Reflection roefficient ($|S_{11}|$). (**b**) RF-to-dc PCE against frequency at different $P_{in}$.

## Results and discussion

Figure 3a compares the simulated reflection coefficient $|S_{11}|$ results of the proposed implantable rectifier and the measured results in free space and the encapsulated minced pork. The -10 dB simulated (measured) fractional percentage BW (FBW) across the major operating frequencies are 4.91% (5.53%), 3.83% (4.34%), and 11.77% (11.44%) for the WiFi white space region. A good simulated (measured) BW is demonstrated by the proposed multiband RF-rectifier, amounting to 90 MHz (100 MHz) for 1.83 GHz, 80 MHz (90 MHz) for 2.10 GHz, 300 MHz (290 MHz) for 2.45 GHz.

**Figure 4:**
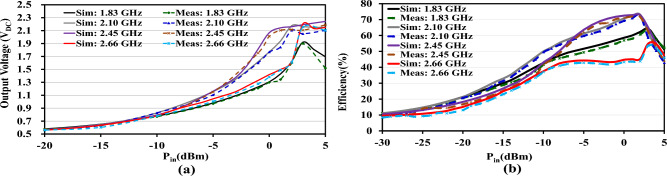
Simulated and measured: (**a**) Output DC Voltage ($V_{DC}$) against $P_{in}$. (**b**) RF-to-dc PCE against $P_{in}$.

**Figure 5:**
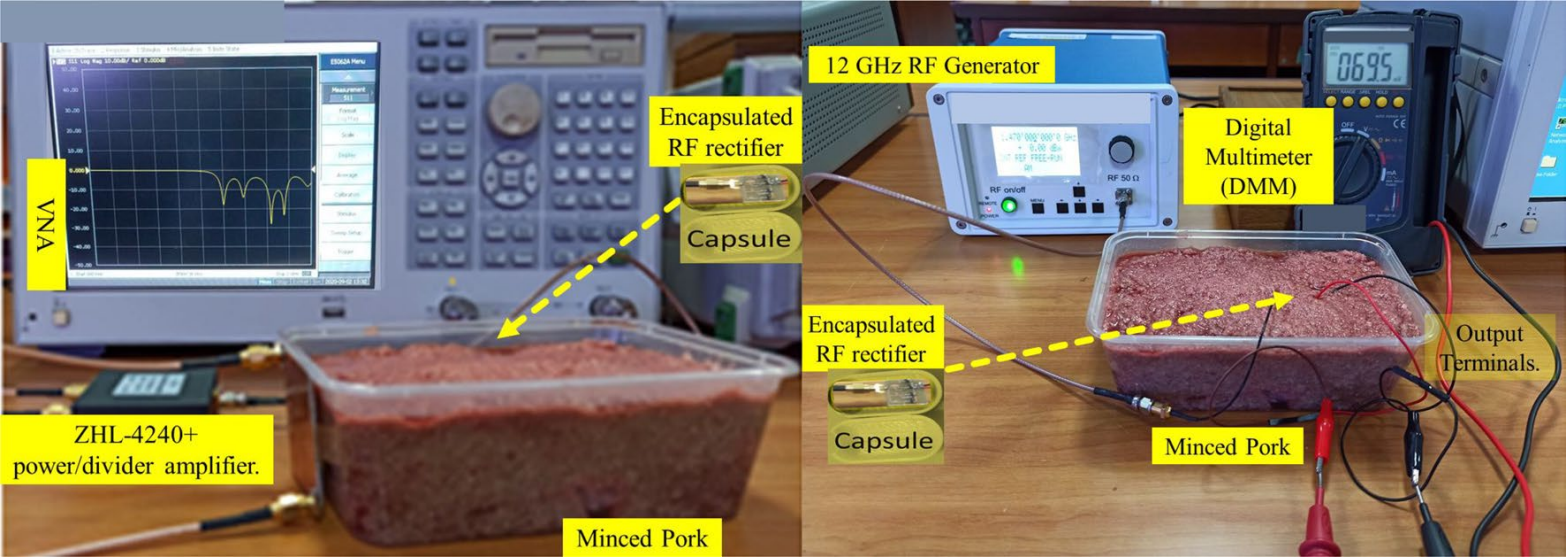
The measurement setups of the proposed RF rectifier prototype.

The RF input power for this test configuration was generated with the help of a 12 GHz signal generator (APSIN12G) and a (ZHL-4240$^+$) power amplifier. The $V_{DC}$ from the measuring equipment is determined using a digital multimeter (DMM) made by *Sanwa* Technology. The relationship between simulated (measured) RF-to-DC PCE against frequency for 0, -10, -20, and -30 dBm is presented in Fig. 3b. RF-to-DC PCE of the proposed RF rectifier is expressed by: $P_{DC}$/$P_{in}$. The $P_{DC}$ total power is calculated by the $V_{DC}$ passing through the load $R_{L}$. At first, there was a deviation in the response of the PCE measurements from the prototype’s fabrication. In comparison to the simulated PCE results at 64.32%, 74.20%, 73.00%, and 54.90% at four operating frequencies ($f_{o}$), 1.83, 2.10, 2.45, and 2.66 GHz, and 2 dBm of input power, respectively, the initial prototype achieved efficiencies of 56.10%, 65.14%, 62.21%, and 44.11%. This could be impacted by losses like the SMA source, part’s accuracy (tolerance), cables, the leads, and TL that interconnect the model’s elements. Also, the dispersed TL, lumped elements’ parasitic capacitance, and phase shift at high frequency can introduce measurement errors. Therefore, the proposed design uses parameter component models to manage the parasitic effect at high frequencies. ADS’s ML optimization and momentum modeling method was used to addressed the problems with the SMA source, connecting lines (TL3 – TL33, TL1 – TL2, and TL), Vias, and other critical parasitic elements. The reactance of the capacitors was obtained from the *muRata* element model to mitigate these constraints. The successive model adjustments led to the final version of the prototype. The measured and simulated findings agree with one another, as shown in Fig. 3a. From Fig. 3b, over 55% RF-to-DC PCE was recorded at 0 dBm, and 11% was also observed at -20 dBm, between 1.80 and 2.55 GHz. The efficiency of the proposed prototype at -20 dBm was investigated to show the design’s significance at low power levels. Hence, at the four operating frequencies, the rectifier reached a maximum PCE of 20.60%, 20.80%, 16.75%, and 13.10%. Figure 4a illustrate the proposed rectifier $V_{DC}$ as a function of input power. Also, Fig. 4b shows how the performance of a simulated and measured RF rectifier changes with the amount of $P_{in}$. For the four operating frequencies, the proposed RF rectifier reached a maximum simulated (measured) RF-to-DC PCE at an input power of 2 dBm at: 64.32% (63.60%), 74.20% (72.70%), 73.00% (72.12%), 55.00% (53.60%). The measurement setups of the proposed RF rectifier prototype is depicted in Fig. 5. There is a slight difference between what was simulated and what was measured. This is because of the effect of component tolerances and the phase shift on the improved prototype. A summary of the simulated and measured results is reported in Table 4.

**Figure 6:**
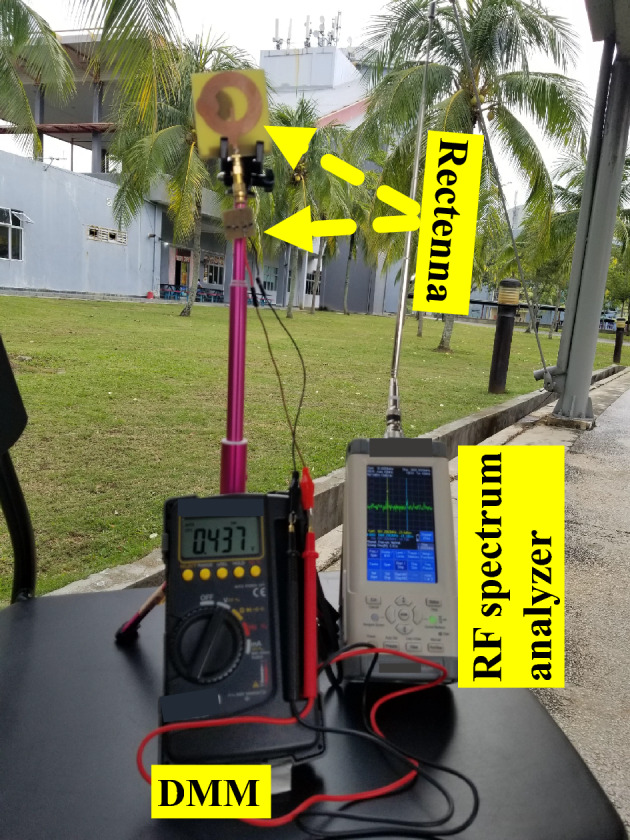
The ambiance measurement setups of the proposed RF rectifier prototype.

In this study, we use a spectrum analyzer to appraise the magnitude of the RF signal captured from different EM sources in the test surroundings. The proposed RF rectifier is subjected to experimental evaluation by utilizing a rectenna system deployed in an open space setting. The rectenna facilitates the evaluation of the RF rectifier within its surrounding ambient environment. Simultaneously, the rectenna serves as an effective means to monitor the RF power’s strength through the analyzer. The evaluation of the rectenna system involved the integration of an RF rectifier with a wideband circular antenna designed to operate efficiently in the frequency range of 1.550 to 3.140 GHz. The antenna exhibited a peak realized gain ranging from 1.93 to 3.20 dB. When utilized in its surrounding environment, the rectenna system successfully generated a $V_{DC}$ with a magnitude of 0.44 V, as depicted in Fig. 6. The findings demonstrate that, under free-space conditions, the RF-to-DC PCE reaches approximately 38.80%.

**Table 4:** Summary of the simulated and measured data.

Parameters	Simulated				Measured (Minced)				Measured (Free space)			
Operating Frequencies	$f_{1}$	$f_{2}$	$f_{3}$	$f_{4}$	$f_{1}$	$f_{2}$	$f_{3}$	$f_{4}$	1	2	3	4
-10 dB BW (MHz)	90	80	300	$f_{3}$	100	90	290	$f_{3}$	90	75	285	$f_{3}$
FBW (%)	4.91	3.83	11.77	$f_{3}$	5.53	4.34	11.44	$f_{3}$	4.91	3.60	11.20	$f_{3}$
PCE: -20 dBm (%)	21.40	21.10	18.26	15.20	20.60	20.80	16.75	13.10	20.94	20.92	17.43	14.12
Peak PCE: 2 dBm (%)	64.30	74.20	73.00	55.00	63.60	72.70	72.10	53.60	63.90	73.22	72.51	54.71

Table 5 shows how the proposed RF rectifier measures up against recent works that have been studied in the literature. While a unique L-shunt $\lambda _{g}$/8 MN technique is employed in this study, it is essential to note that the proposed design has improved operational BW, lower electrical length and higher performance at low input power. The authors of^20,21^, and^22^ recorded a single-band rectifier that is fairly narrow. The proposed design record a good FBW of 11.44% across the white space ISM band. Increased PCE at a high $P_{in}$ of 12 dBm was shown by the authors in^23^. Along with the authors’ illustration of high $P_{in}$ (12 and 5 dBm) in^24^ and^25^, respectively the longer electrical lengths also exhibit low RF-to-DC PCE. The authors in^26,27^, and^8^ demonstrate a multiband RF rectifier that operates at low $P_{in}$ between -5 and 5 dBm, at the cost of considerable electrical length. At 2 dBm, our proposed design achieved 72.7% RF-to-DC PCE. Similarly, the works reported by the authors in^29–31^, and^32^, a common approach in the reported works involves the implementation of a dual single diode with a narrow band and a large electrical size. However, this method presents challenges, such as complex circuitry and a high input power requirement to ensure the circuit’s efficient functioning within implants. These design choices reduce overall efficiency and limit the harnessing of available RF power at specific frequencies, as stated in^29^ at 3.50 GHz and in^32^ at 3.50, 4.90, and 5.80 GHz. Compared to the work shown in the related literature, the proposed method demonstrates improved compactness and has a shorter electrical length of 0.27$\lambda _{g}$ $\times$ 0.29$\lambda _{g}$. Between 1.80 and 2.55 GHz, the proposed topology reached over 55% RF-to-DC PCE at 0 dBm. For low-power applications, this work shows an enhanced performance of 20.60%, 20.80%, 16.75%, and 13.10% at -20 dBm for each of the four frequencies, respectively.

**Table 5:** A comparison between the proposed RF rectifier and the recent implantable rectifiers.

**[Ref.]**	$f_{o}$ **(GHz)**	**Dimension** $\lambda _{g}$ $\times$ $\lambda _{g}$	**Max. PCE** (%) : [$P_{in}$ (dBm)]	**Diode/ Topology**	**Substrate** ($\varepsilon _{r}$)
^20^	0.915	NA	59.70 : [30]	SMS7630 Voltage Multiplier	RO3210 (10.2)
^21^	0.673	NA	40 : [NA]	HSMS285B Voltage Multiplier	FR-4 (4.4)
^22^	0.915	0.14 x 0.16	51.7 : [-5]	HSMS2852 Dual- Single Series	RO3210 (10.2)
^23^	0.915, 2.45	0.36 x 0.20	81.7, 73.1 : [12]	HSMS2852 Dual-Single Series	Arlon AD 255 (2.55)
^24^	2.45, 5.8	1.37 x 0.83	63.00, 54.80 : [12]	HSMS2850 SSr	FR-4 (4.4)
^25^	2.32, 3.48	0.46 x 0.27	14.5, 64.2 : [5]	SMS7621 Single-Shunt	TACONIC TLY-5 (2.2)
^26^	1.78, 2.35, 4.97	NA	60.95, 71.35 ; 54.01 : [5]	BAT15-03W Single-Shunt	RO4003C (3.38)
^27^	1.85, 2.15, 2.48	0.41 x 0.74	48, 52, 45 : [-5]	HSMS2850 SSr	RO5880 (2.2)
^28^	1.95, 2.7, 5.8	0.43 x 0.27	62.2, 59.40, 48.9 : [0]	HSMS2860 SSr	RO4003 (3.38)
^29^	1.80, 2.10, 2.40 2.65, 3.50	0.75 $\times$ 0.75	23.2 : [-20]	SMS7630 SSr	F4BM-2 (2.65)
^30^	0.90, 1.80, 2.10, 2.40	NA	15 : [-20]	MSS20-141 Dual-Single Series	R04003 (3.3)
^31^	0.55, 0.75, 0.9 1.85, 2.15, 2.45	0.1 $\times$ 0.11	67 : [-5]	SMS7630 Dual-Single Series	RO5880 (2.2)
^32^	1.80, 2.10, 2.40 2.60, 3.50, 4.90, 5.80	0.50 $\times$ 0.40	44.40, 43.90, 45.40 43.40, 36.10, 32.40 28.30 : [-10]	SMS7630 SSr	RO5880 (2.2)
This Work	1.81, 2.03, 2.45, 2.63	0.27 $\times$ 0.29	63.6, 72.7, 72.10, 53.6 : [2]	HSMS2850 SSr	FR-4 (5.4)

SSr: Single Series Diode

$\lambda _{g}$: Wavelength at the lowest operating frequency ($f_{o}$), NA: Not available.

## Conclusion

This study explores a unique RF rectifier design that uses an improved L-shunt $\lambda _{g}$/8 using a sequential matching technique. A series inductor and a radial stub incorporated into a distributed MN is applied to match the impedance of the proposed SSr RF rectifier across the four cells. The technique enhanced the design’s BW and compactness and demonstrated its ability to efficiently leverage the frequency domain by employing multi-band operation and displaying a good impedance bandwidth. The proposed multiband RF rectifier prototype realized a simulated (measured) -10 dB BW of 90 MHz (100 MHz) for 1.83 GHz, 80 MHz (90 MHz) for 2.10 GHz, 300 MHz (290 MHz) for 2.45 GHz and FBW of 4.91% (5.53%), 3.83% (4.34%), and 11.77% (11.44%), respectively. The proposed design architecture produced an output $V_{DC}$ of 1.61 V and a high PCE of 72.7%. The RF rectifier’s measurements on the PCB board were 0.27$\lambda _{g}$ $\times$ 0.29$\lambda _{g}$. Hence, if managed carefully, the proposed design has practical applications in biomedical engineering, opening up possibilities for powering a wide range of implantable medical devices using harvested RF energy.

## Data Availability

All data generated or analysed during this study are included in this published article.
